# Primary malposition of peripherally inserted central catheter in the inferior vena cava among neonates: a case series

**DOI:** 10.3389/fped.2026.1784844

**Published:** 2026-03-25

**Authors:** Ruiqing Song, Shujing Jia, Peihao Wang, Jingjing Zhao, Junge Li, Kun Wang

**Affiliations:** 1 Department of Neonatology, The Third Affiliated Hospital of Zhengzhou University, Zhengzhou, China; 2 Department of Nursing, The Third Affiliated Hospital of Zhengzhou University, Zhengzhou, China

**Keywords:** catheterization, inferior vena cava, malpositions, neonates, PICC

## Abstract

**Introduction:**

Lower-extremity insertion of peripherally inserted central catheters (PICCs) is recommended in neonates to reduce tip malposition risk, yet primary malposition into the inferior vena cava (IVC) remains underreported. This study examined the incidence, contributing factors, and management outcomes of this complication in a neonatal intensive care unit (NICU).

**Methods:**

We conducted a retrospective case series in a tertiary Chinese hospital NICU (February 2022–March 2024). Among 18 neonates with confirmed IVC malposition after lower-extremity PICC placement, we assessed tip location by post-insertion x-ray, collected clinical and catheterization data, and analyzed associations between treatment strategies and final tip position.

**Results:**

18 of 723 lower-extremity PICC insertions (2.5%) showed primary IVC malposition. Concurrent umbilical venous catheter, respiratory support, and patient positioning were associated factors. We classified malpositions as high (T9–T12, *n* = 11) or low (L2–S2, *n* = 7). External manual repositioning corrected 90.9% of high malpositions. Catheter withdrawal by 4–8 cm with re-advancement succeeded in 85.7% of low-level cases. All catheters were retained throughout therapy.

**Conclusion:**

Primary IVC malposition is linked to positioning, elevated intra-abdominal pressure, and umbilical catheter presence. Elevating the infant's hips during insertion can promote proper catheter ascent. If resistance occurs, removing an indwelling umbilical catheter often resolves malposition. Ultrasound-guided assessment supports successful correction in most cases, emphasizing preventive measures and tailored management.

## 1 Introduction

PICCs provide crucial long-term venous access for critically ill neonates, especially very low birth weight infants, by delivering essential nutrients and medications (1). Compared with peripheral venous catheters, PICCs offer significant advantages, including fewer venipunctures, reduced procedural pain, longer indwelling time, and greater safety and efficacy (2, 3). However, their use is associated with potential complications such as catheter occlusion, rupture, infection, and thrombosis.

Catheter tip malposition is the most common of these complications, with a reported incidence of up to 48.2% (4). Primary malposition, occurring during or immediately after catheter insertion, is a significant factor leading to serious adverse events such as venous thrombosis, pleural effusion, and phlebitis (5–7). The safety and efficacy of PICCs depend largely on correct tip placement, which helps reduce complications and prolong catheter retention time (8–10). Accordingly, the Infusion Nurses Society (INS) guidelines (8) recommend that the tip of a central venous catheter be positioned in the superior vena cava or the inferior vena cava at the junction of the right atrium; malposition is defined as the catheter tip erroneously entering non-target branches such as hepatic veins, renal veins, ascending lumbar veins, or the contralateral iliac vein.

To minimize the risk of PICC malposition, the National Association of Neonatal Nurses (NANN) designates the lower extremity as the recommended site for catheter insertion in neonates (11). This recommendation is supported by several studies indicating a lower incidence of malposition with lower-extremity insertions compared with upper-extremity or scalp vein insertions (12–14).

Although evidence suggests that lower-extremity placement of PICCs may reduce the risk of catheter malposition in neonates, this approach carries a specific and underrecognized complication: primary malposition of the PICC in the inferior vena cava. Although uncommon, this malposition can lead to serious consequences, including catheter dysfunction, nerve injury, and abdominal wall-related complications (15). These outcomes threaten patient safety and pose significant challenges for clinicians. Early identification of risk factors, along with targeted preventive measures and timely correction, is therefore essential for ensuring infusion safety, reducing complications, shortening catheter indwelling time, and avoiding unnecessary healthcare resource utilization.

However, there is currently limited awareness among neonatologists regarding lower-extremity PICC malposition, and published reports on the subject are limited. Thus, we conducted a retrospective case series of neonates who developed primary PICC malposition in the inferior vena cava between February 2022 and March 2024. Our study systematically describes its incidence, clinical features, management, and outcomes, to inform early recognition and standardized management strategies for primary PICC malposition in the inferior vena cava.

## 2 Materials and methods

This retrospective case series was conducted in the Neonatal Intensive Care Unit (NICU) of a tertiary hospital in China. We screened all neonates who had PICC placements from February 2022 to March 2024. Inclusion was based on the initial post-insertion x-ray confirming primary PICC tip malposition in the inferior vena cava. Our data were extracted from the hospital's electronic medical records and the departmental central venous catheter maintenance database. A standardized data collection form was used to capture comprehensive baseline characteristics for each infant, including gender, gestational age, weight at catheter insertion, ventilation mode, presence of an umbilical venous catheter (UVC), insertion site, insertion vein, malposition location, and indwelling time.

### 2.1 Diagnosis

In this study, the assessment of PICC tip location was primarily based on radiographic findings. Immediately after PICC insertion, we obtain a routine x-ray to confirm the catheter tip position. We then classified PICC tip location into three categories: optimal, non-ideal but acceptable, and malpositioned. We defined the optimal position as the lower third of the IVC near the cavoatrial junction. We considered that a tip position slightly above or below the ideal location, while not optimal, may still be clinically acceptable as long as the catheter functions well and no complications arise. Primary malposition of the PICC in the inferior vena cava was defined as a tip position located in non-target branches. To ensure the accuracy of key imaging interpretations, all relevant radiographs were independently reviewed by a radiologist and a certified intravenous therapy nurse. Both reviewers were blinded to each other's initial assessments and to the clinical details of the cases. After their independent reviews, the assessments were compared. Inter-rater reliability was calculated using Cohen's kappa coefficient. For the 18 cases included in this series, there was 100% agreement (*κ* = 1.0) on the primary outcome that the tip was in a true malpositioned location. However, for the specific description of the malposition, there was minor descriptive variability in 2 cases (11.1%), which was resolved through subsequent consensus discussion between the two reviewers, involving a senior neonatologist as a third reviewer if necessary.

### 2.2 Interventions

Once we confirm PICC tip malposition, the operator should correct it immediately. In our study, we employed two distinct approaches to manage primary PICC malposition within the inferior vena cava: (1) The external manual method: The infant was placed in a supine position with the hips elevated. An assistant then gently tapped the abdominal wall near the estimated location of the catheter tip. Concurrently, a pulsatile saline flush was administered to facilitate the movement of the catheter tip toward the upper segment of the IVC under gravity. (2) The conventional method: The indwelling catheter was first slowly withdrawn by 5–8 cm, then a pulsatile saline flush was performed during slow re-advancement of the catheter to the intended length.

### 2.3 Ethics

The study protocol was reviewed and approved by the Ethics Committee of the Third Affiliated Hospital of Zhengzhou University (Approval No. 2024-017-01).

## 3 Results

### 3.1 Patient general characteristics

During the study period, primary PICC malposition into the inferior vena cava was identified in 18 of the 723 neonates who underwent lower-extremity insertion, resulting in an incidence of 2.5%. The cohort comprised 9 males and 9 females. The mean gestational age was 29.55 ± 3.29 weeks (range: 25⁺⁶–37 weeks). The mean body weight at insertion was 1.31 ± 0.64 kg, and the mean postnatal age was 8.12 ± 8.64 days. At the time of catheterization, 6 infants (33.3%) were on mechanical ventilation, and 12 (66.7%) received nasal continuous positive airway pressure (nCPAP). A UVC was still in place in 10 neonates (55.6%) at the time of PICC insertion, while it had been removed in the remaining 8 (44.4%). The right lower limb was the predominant insertion site (*n* = 13, 72.2%), with the left lower limb used in 5 cases (27.8%). The great saphenous vein was the most common puncture site (*n* = 15, 83.3%), followed by the small saphenous vein (*n* = 2, 11.1%) and the femoral vein (*n* = 1, 5.6%). Patient General characteristics are summarized in Table 1.

**Table 1:** Characteristics of the 18 neonates with PICC malposition.

Case	Gender	Gestational age	Weight (kg)	Ventilation mode	UVC removed	Abdominal condition	Insertion site	Insertion vein	Malposition site	Indwelling time (days)
N1	Male	30^+3^	1.16	Non-invasive ventilator	No	Flat and soft	right lower limb	Great saphenous vein	Retroflexion downward at T12	18
N2	Female	30^+1^	0.97	Non-invasive ventilator	No	Flat and soft	right lower limb	Great saphenous vein	Retroflexion to the left at T12	15
N3	Male	26^+5^	1.6	Non-invasive ventilator	Yes	Flat and soft	Right lower limb	Small saphenous vein	Retroflexion to the right at T10–T11	13
N4	Male	29^+5^	0.85	Invasive ventilator	No	Flat and soft	Right lower limb	Great saphenous vein	Retroflexion to the right at T11	20
N5	Female	28^+5^	1.1	Non-invasive ventilator	No	Flat and soft	Right lower limb	Great saphenous vein	Retroflexion to the right at T12	13
N6	Male	37	2.8	Invasive ventilator	Yes	Flat and soft	Right lower limb	Great saphenous vein	Retroflexion downward at L3	14
N7	Male	27^+6^	0.72	Invasive ventilator	No	Flat and soft	Right lower limb	Great saphenous vein	Retroflexion downward at T9	20
N8	Male	31^+2^	1.67	Non-invasive ventilator	Yes	Flat and soft	Right lower limb	Great saphenous vein	Catheter looping at L5	25
N9	Female	29^+1^	1.23	Non-invasive ventilator	Yes	Flat and soft	Left lower limb	Great saphenous vein	Retroflexion downward and looping at L2–L3	15
N10	Female	31^+3^	1.4	Non-invasive ventilator	Yes	Flat and soft	Right lower limb	Small saphenous vein	Retroflexion downward at T9	12
N11	Female	27	0.8	Non-invasive ventilator	No	Flat and soft	Left lower limb	Femoral vein	Retroflexion downward at T12	9
N12	Female	34	2	Invasive ventilator	Yes	Flat and soft	Left lower limb	Great saphenous vein	Retroflexion to the left at L3	7
N13	Female	27	0.9	Non-invasive ventilator	No	Flat and soft	Right lower limb	Great saphenous vein	Retroflexion downward and looping at L3	12
N14	Male	29^+1^	1.1	Non-invasive ventilator	Yes	Distended	Right lower limb	Great saphenous vein	Retroflexion downward at L2	14
N15	Male	26^+1^	0.8	Non-invasive ventilator	No	Flat and soft	Right lower limb	Great saphenous vein	Retroflexion to the right at T11	13
N16	Male	26^+2^	0.9	Non-invasive ventilator	No	Flat and soft	Left lower limb	Great saphenous vein	Retroflexion downward at T12	13
N17	Female	25^+6^	0.7	Invasive ventilator	No	Flat and soft	Right lower limb	Great saphenous vein	Reverse folded to the right at T11	26
N18	Female	36^+6^	2.9	Invasive ventilator	Yes	Flat and soft	Left lower limb	Great saphenous vein	Retroflexion downward and looping at S2	8

### 3.2 Outcomes of the different management approaches

All 18 cases of primary IVC malposition were successfully managed, with every catheter retained for the full course of intravenous therapy. In 11 cases, the PICC initial malpositiontip was below the diaphragm, within the T9–T12 range. We used the external manual repositioning method and successfully moved the tip to about 0–1 cm above the diaphragm in 10 of them (90.9%). In the one case where it failed, successful repositioning was achieved by withdrawing the catheter by 4 cm, followed by a standard pulsed flush. For the 7 cases with the catheter tip in the S2-L5 region, the external manual repositioning failed. Subsequent withdrawal of the catheter by 5–8 cm, followed by re-advancement, successfully repositioned 6 catheters (85.7%). The remaining case (14.3%) required conversion to a midline catheter. Detailed outcomes are presented in Table 2.

**Table 2:** Management approaches for primary PICC malposition in the inferior vena cava (*n* = 18).

Malposition site	Cases	Management approach	Outcome
Retroflexion downward at T9–T12	5	External manual repositioning successful	Tip located in the upper segment of the inferior vena cava
Rightward retroflexion at T9–T12	5	External manual repositioning was successful in 4 cases; 1 case: catheter withdrawn 4 cm, infant placed supine with hips elevated, catheter advanced with simultaneous saline flush	Tip located the upper segment of the inferior vena cava
Leftward retroflexion at T9–T12	1	External manual repositioning successful	Tip located in the upper segment of the inferior vena cava
Downward retroflexion at L2–S2	5	4 cases: catheter withdrawn 5–7 cm and successfully re-advanced; 1 case: repositioning failed, catheter used as a midline catheter	4 cases: tip in upper IVC; 1 case: used as midline catheter
Leftward retroflexion at L3	1	Catheter withdrawn 5 cm, infant placed supine with hips elevated, catheter advanced with simultaneous saline flush	Tip located in the upper segment of the inferior vena cava
Catheter looping at L5	1	Catheter withdrawn 8 cm, infant placed supine with hips elevated, catheter advanced with simultaneous saline flush	Tip located in the upper segment of the inferior vena cava

Based on these findings, we suggest that operators choose a repositioning method based on the location and type of malposition to improve success rates. For cases where the tip malposition occurs between T9 and T12, we recommend first trying the external manual method. For those with tip malposition between S2 and L5, the conventional method is a better choice.

## 4 Discussion

In our study, the incidence of primary PICC malposition following lower-extremity insertion was 2.5%. We attribute this low incidence to both stringent insertion protocols and consistent radiographic interpretation. Our unit follows a standardized PICC insertion protocol with operators trained in tip positioning techniques, which likely minimizes true malpositions. Simultaneously, as we have now documented, our radiographic interpretation process demonstrates good inter-observer reliability, reducing the likelihood of misclassification.

Our analysis suggests that primary PICC malposition into the inferior vena cava is influenced by a combination of factors, namely the patient's position during insertion, increased intra-abdominal pressure, occupancy by a UVC, and anatomical variations in local vasculature.

Previous studies have established that patient positioning significantly affects PICC tip location (12). In our cohort, we observed that all 18 malpositioned PICCs had been inserted with the infant in a standard supine position. However, in 17 of these infants, successful correction was achieved simply by elevating the hips during a pulsatile flush, a maneuver that effectively guided the tip to the ideal position within the inferior vena cava.

**Figure 1:**
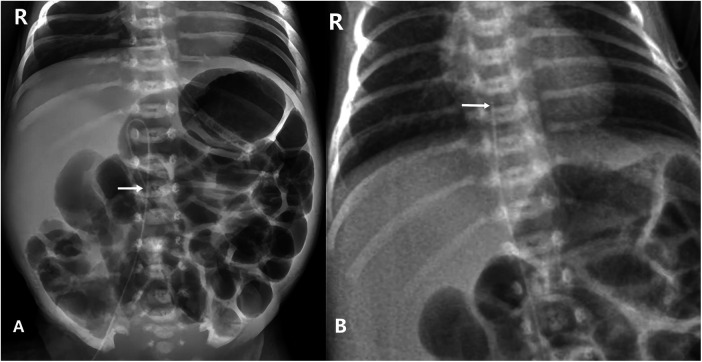
Plain chest and abdominal X-rays show: **(A)** Pre-repositioning: the PICC lies below the diaphragm, it courses downward from the T11–T12 level, with its tip lying at L2–L3. **(B)** Post-repositioning: the PICC tip lies at T7–T8, approximately 0.5 cm above the diaphragm.

The association between PICC malposition and mechanical ventilation, as reported by Grasso et al. (16), along with the established link to elevated intra-abdominal pressure (17), may be explained by the following mechanism in neonates: ventilator support coupled with crying can increase intra-abdominal pressure. This pressure compresses the inferior vena cava, disrupting venous flow and directing the catheter into a retroflexed or aberrant path (Figure 1). While we recognize that elevated intra-abdominal pressure is likely a key factor. However, because this was a retrospective study, we did not collect abdominal circumference measurements in our past clinical records—measurements that could have served as a useful surrogate marker for changes in intra-abdominal pressure. Future studies should include such measurements, ideally taken in real time during PICC insertion, to quantitatively assess the relationship between intra-abdominal pressure, respiratory support mode, and the risk of catheter malposition.

**Figure 2:**
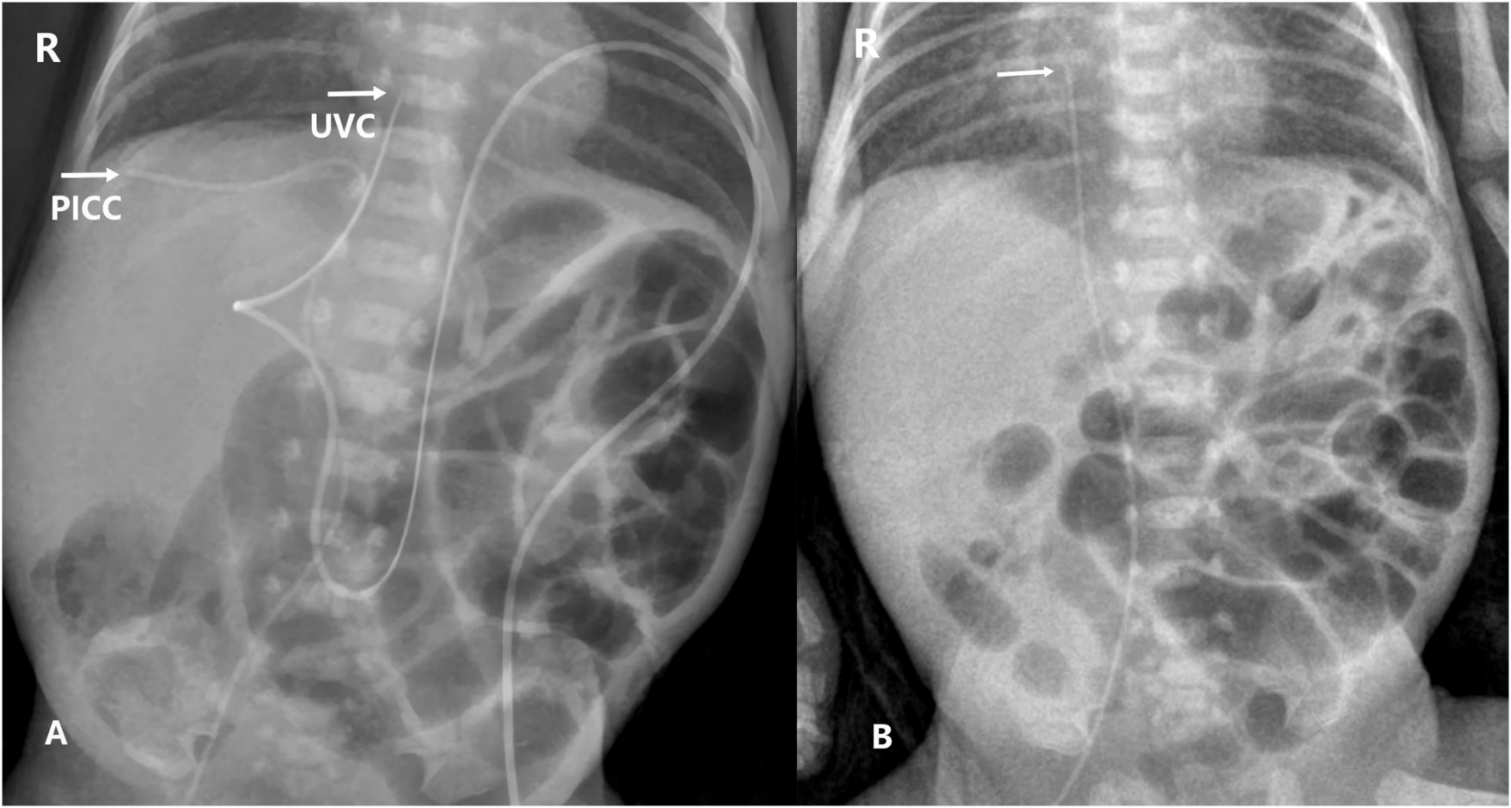
Plain chest and abdominal X-rays show: **(A)** Pre-repositioning: the UVC tip at T9. the PICC courses into an intrahepatic vein below the diaphragm (T10–11), with the tip terminating within the liver. **(B)** Post-repositioning: the PICC tip at T7–8, about 1 cm above the diaphragm.

Elabbasy et al. reported a strong association between PICC malposition and the presence of a functioning UVC (18). The proposed mechanism is that the UVC occupies space within the inferior vena cava, increasing resistance to PICC advancement and thereby predisposing it to malposition. This is consistent with our findings: among the 11 cases of catheter looping at T9–T12, 9 (81.8%) had a UVC *in situ* at the time of PICC insertion (Figure 2). Regarding the coexistence of a UVC and PICC, in our unit, as in many NICUs, UVCs are often placed immediately after birth for emergent access. The decision to remove a UVC before PICC insertion is not always straightforward. Clinicians may opt to leave a functioning UVC *in situ*, especially in extremely low birth weight infants with tenuous access, until the PICC placement is confirmed and operational, to avoid a period without central access. This practice, while ensuring continuous vascular access, inadvertently creates a competing space within the IVC, increasing resistance and predisposing the advancing PICC to malposition, as our data suggests. Notably, in all 9 cases, the malposition was successfully corrected using external manual repositioning after UVC removal. While this suggests a clear clinical sequence, the efficacy of this specific intervention requires further validation through larger, prospective studies.

Vascular anomalies are a recognized factor that can alter PICC catheter trajectory (19). We encountered one illustrative case where the catheter tip coiled in the external iliac vein and repositioning failed. Subsequent ultrasound identified a venous stenosis. Such stenotic or malformed vessels can physically obstruct catheter advancement or divert it into an unintended path. Additionally, these anatomical variations may alter local hemodynamics, further elevating the risk of malposition. It is worth noting that we did not routinely use bedside ultrasound for vessel assessment before PICC insertion or during repositioning in this study. However, we now recommend using ultrasound for vessel assessment and real-time guidance during PICC placement to improve insertion success and reduce the risk of malposition.

While x-ray based on vertebral or cardiac landmarks is the conventional method for PICC tip verification (20, 21), its accuracy is increasingly questioned (22–24). Real-time ultrasound offers a compelling alternative with higher sensitivity and specificity. Utilizing ultrasound for tip location reduces the number of x-rays, thereby cutting costs and eliminating radiation exposure, all while improving accuracy (25). This is especially advantageous for PICCs inserted from the lower extremity veins, where x-ray interpretation is more challenging (26, 27).

Our study also has several limitations. Its retrospective design inherently limits the strength of causal inferences and relies on the accuracy and completeness of existing medical records. Our findings should be interpreted as hypothesis-generating rather than definitive, and prospective validation is warranted in the future. In addition, its modest sample size limited the statistical power for subgroup analyses. Moreover, the assignment of repositioning methods was not randomized but was based on the catheter's position. This non-randomized approach may have introduced selection bias into the comparison of success rates between methods, potentially limiting the generalizability of our findings to other clinical settings.

## 5 Conclusion

Primary PICC malposition in the inferior vena cava, while uncommon, is a clinically significant challenge in neonatal care. Our findings confirm its strong association with patient position during insertion, elevated intra-abdominal pressure, the presence of a concurrent UVC, and individual vascular anatomy. A proactive clinical strategy is therefore recommended. Before lower-extremity PICC insertion, this entails ensuring optimal patient positioning, monitoring for factors that increase intra-abdominal pressure, and the timely removal of unnecessary UVCs to mitigate malposition risk.

## Data Availability

The original contributions presented in the study are included in the article/Supplementary Material, further inquiries can be directed to the corresponding author.
